# Higher EpCAM-Positive Extracellular Vesicle Concentration in Ascites Is Associated with Shorter Progression-Free Survival of Patients with Advanced High-Grade Serous Carcinoma

**DOI:** 10.3390/ijms25126780

**Published:** 2024-06-20

**Authors:** Maruša Herzog, Ivan Verdenik, Borut Kobal, Katarina Černe

**Affiliations:** 1 Division of Gynecology and Obstetrics, University Medical Centre Ljubljana, SI-1000 Ljubljana, Slovenia; marusa.herzog@kclj.si (M.H.); ivan.verdenik@guest.arnes.si (I.V.); borut.kobal@kclj.si (B.K.); 2 Department of Gynecology and Obstetrics, Faculty of Medicine, University of Ljubljana, SI-1000 Ljubljana, Slovenia; 3 Institute of Pharmacology and Experimental Toxicology, Faculty of Medicine, University of Ljubljana, SI-1000 Ljubljana, Slovenia

**Keywords:** EpCAM, extracellular vesicles, HGSC (ovarian cancer), prognosis, biomarkers, personalized medicine

## Abstract

Platinum-resistant high-grade serous carcinoma (HGSC) is an incurable disease, so biomarkers that could help with timely treatment adjustments and personalized approach are extensively being sought. Tumor-derived extracellular vesicles (EVs) that can be isolated from ascites and blood of HGSC patients are such promising biomarkers. Epithelial cell adhesion molecule (EpCAM) expression is upregulated in most epithelium-derived tumors; however, studies on prognostic value of EpCAM overexpression in ovarian carcinoma have shown contradictory results. The aim of our study was to evaluate the potential of total and EpCAM-positive EVs as prognostic and predictive biomarkers for advanced HGSC. Flow cytometry was used to determine the concentration of total and EpCAM-positive EVs in paired pretreatment ascites and plasma samples of 37 patients with advanced HGSC who underwent different first-line therapy. We found that higher EpCAM-positive EVs concentration in ascites is associated with shorter progression-free survival (PFS) regardless of treatment strategy. We also found a strong correlation of EpCAM-positive EVs concentration between ascites and plasma. Our findings indicate that EpCAM-positive EVs in ascites of patients with advanced HGSC have the potential to serve as prognostic biomarkers for predicting early recurrence and thereby likelihood of more aggressive tumor biology and development of chemoresistance.

## 1. Introduction

Ovarian cancer is the leading cause of death among gynecological cancers in developed countries [1,2]. More than 90% of malignant ovarian tumors are of epithelial origin, and high-grade serous carcinoma (HGSC) is the most common and most lethal type [3]. Despite efforts, overall survival of patients with HGSC has not significantly changed in recent decades. There are still no validated predictive biomarkers for platinum resistance or resistance to targeted therapies or prognostic biomarkers in clinical practice that could help inform treatment decisions and enable more personalized treatment [3]. An individualized approach would require the identification of appropriate molecular targets in tumor tissue, but tissue biopsies are invasive and carry potential risk for patients. Perhaps even more importantly, because of the genetic and cellular heterogeneity of HGSC, tissue biopsies might not be fully representable [4]. To overcome this, the potential of extracellular vesicles (EVs) that can be obtained by liquid biopsy of ascites or blood is being extensively studied [5,6]. In vitro studies on EVs derived from ovarian cancer cell lines show that EVs promote tumor growth, metastasis, immune evasion, and development of chemoresistance [4,7,8,9,10]. Furthermore, the composition of EVs reflects the (patho)physiological state of the cell of origin [4,5], which makes them a promising source of diagnostic, prognostic, and predictive biomarker candidates. In ovarian cancer, the concentration of total EVs is typically higher, but clinical studies on EVs potential as diagnostic biomarkers have implied that determining total EVs concentration might not be sufficient [11,12,13]. In addition, none of these studies has evaluated the predictive or prognostic value of total EVs. Determining tumor-specific vesicular cargo (e.g., miRNA and protein content) can improve the potential of EVs as biomarkers [11,12,13].

One of the most studied EVs’ cargo proteins in ovarian cancer is epithelial cell adhesion molecule (EpCAM, also known as CD326), a transmembrane glycoprotein involved in cell proliferation and differentiation [14]. In healthy tissue, EpCAM plays a role in the process of morphogenesis, tissue regeneration, and stem cell maintenance, whereas in cancer, its overexpression has been found to promote tumor progression [14]. The proposed mechanism is negative regulation of cell adhesion, consequential loss of cell polarity, and contact inhibition, which lead to uncontrolled proliferation and migration of tumor cells [14]. Increased EpCAM levels are typical for epithelium-derived tumors, and EpCAM overexpression in tumor tissue has been associated with poor prognosis in breast, pancreatic, urothelial, and gallbladder carcinoma [14,15,16,17]. In renal and thyroid carcinoma, on the other hand, EpCAM overexpression was associated with increased survival [14]. In ovarian cancer, studies on EpCAM overexpression in tumor tissue and survival have shown contradictory results [18,19,20]. To further elucidate the potential of EpCAM as a prognostic biomarker in ovarian cancer, our research group focused on EpCAM-positive EVs in ascites and plasma. Moreover, the determination of EpCAM-positive EVs in clinical samples might be helpful for evaluation of the potential of EpCAM-targeted therapy and could explain the disparate results concerning its effectiveness [21,22,23].

To date, only a small number of studies of EpCAM-positive EVs has been conducted on clinical samples of ascites [13,24] or plasma [11,25,26,27] of ovarian cancer patients. None of these studies has evaluated the predictive or prognostic value of EpCAM-positive EVs.

The aim of our study was to evaluate the potential of total and EpCAM-positive EVs as prognostic and predictive biomarkers for advanced HGSC that could help select patients who would benefit from anti-EpCAM targeted therapy and provide an improved personalized approach. Fluorescence-triggered flow cytometry (FT-FCM) was used to determine total and EpCAM-positive EVs’ levels in paired ascites and plasma samples of patients with advanced HGSC. To our knowledge, this is the first study to evaluate the association of total and EpCAM-positive EVs in paired pretreatment samples of ascites and plasma with clinical characteristics, treatment outcome, and prognosis of patients with advanced HGSC.

## 2. Results

### 2.1. Patients’ Characteristics

Characteristics of the 37 patients with advanced HGSC are presented in Table 1.

### 2.2. Correlation of Total and EpCAM-Positive EVs Concentration between Ascites and Plasma

**Figure 1:**
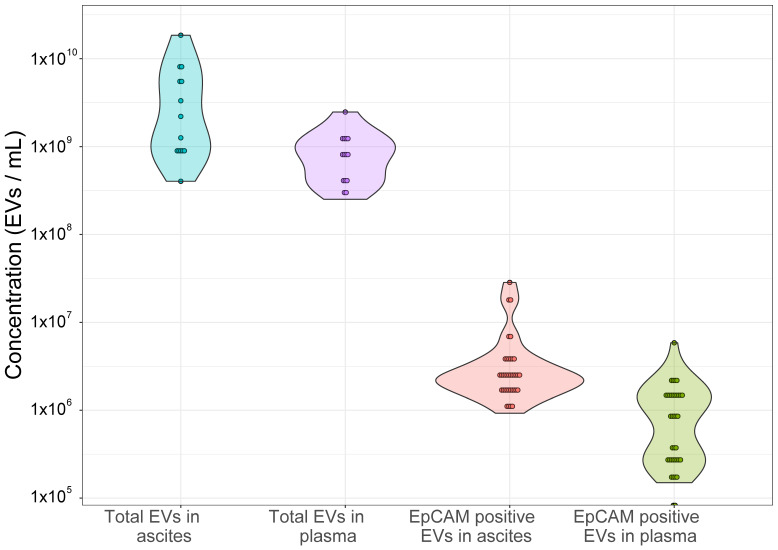
Total and EpCAM-positive EVs concentration in ascites and plasma of patients with advanced HGSC (N = 14 for total and N = 37 for EpCAM-positive EVs). The concentration of EVs in 20 μL of platelet-poor plasma and ascites samples was measured with fluorescence-triggered flow cytometry using volumetric measurement (events/mL). To calculate the number of EV per mL in body fluids (plasma and ascites), the dilution factor during sample preparation and staining was considered. Total (calcein-positive) EVs concentration in ascites and plasma was determined in 14 patients and EpCAM-positive EVs concentration in ascites and plasma in 37 patients with advanced HGSC.

Using FT-FCM, total EVs and EpCAM-positive EVs concentration in ascites and plasma was determined. Median value (25th–75th percentiles) for total EVs in ascites was 1.73 × 10^9^ (9.16 × 10^8^–6.38 × 10^9^)/mL and in plasma 7.65 × 10^8^ (3.95 × 10^8^–1.20 × 10^9^)/mL. For EpCAM-positive EVs, median value (25–75 centiles) in ascites was 2.28 × 10^6^ (1.67 × 10^6^–3.37 × 10^6^)/mL and in plasma 7.50 × 10^5^ (2.50 × 10^5^–1.48 × 10^6^)/mL (Figure 1).

**Figure 2:**
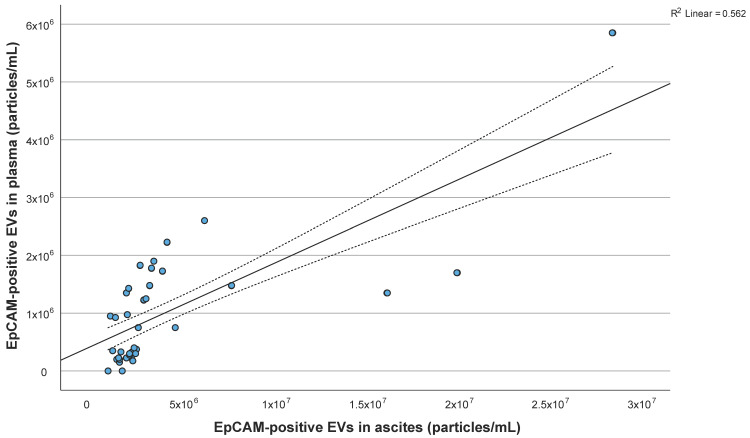
Correlation of EpCAM-positive extracellular vesicles (EVs) concentration between ascites and plasma. The upper and lower dashed lines on either side of the linear regression line represent the 95% confidence interval (CI).

No significant correlation of total (calcein-labeled) EVs concentration between ascites and plasma was found (*p* = 0.345). On the other hand, there was significant correlation of EpCAM-positive EVs concentration between ascites and plasma (r = 0.749, *p* < 0.001; Figure 2). EpCAM-positive EVs concentration in ascites was significantly higher than in plasma (*p* < 0.001).

### 2.3. Total and EpCAM-Positive EVs Concentration and Clinical Characteristics, Treatment Outcome, and Prognosis of Patients with Advanced HGSC

We evaluated the relationship between total and EpCAM-positive EVs concentration in pretreatment plasma and ascites samples and response to therapy, progression-free survival (PFS), and overall survival (OS). First, we compared the two groups of patients, 22 with primary inoperable disease and 15 with primary cytoreductive surgery. No significant difference in total or EpCAM-positive EVs concentration in ascites (*p* = 0.318 and *p* = 0.262) or plasma (*p* = 0.805 and *p* = 0.098, respectively) between the two groups was observed. Importantly, there was also no significant difference in PFS (HR = 1.712, 95% CI: 0.824–3.558, *p* = 0.150) or OS (HR = 1.504, 95% CI: 0.694–3.259, *p* = 0.301) between the two groups. For the 15 patients that had primary cytoreductive surgery, correlation between resection success (residual disease R0, R1, or R2) and concentration of total and EpCAM-positive EVs was assessed. There was no significant correlation of resection success at primary cytoreductive surgery and total or EpCAM-positive EVs concentration in pretreatment ascites (*p* = 1000 and *p* = 0.732) or plasma (*p* = 0.403 and *p* = 0.870, respectively), though. Of the 22 patients with primary inoperable disease, only 16 were eligible for interval debulking surgery (IDS). For these 16 patients, the correlation of chemotherapy response score (CRS) with total and EpCAM-positive EVs was observed. No significant correlation between CRS and total or EpCAM-positive EVs concentration in ascites (*p* = 0.908 and *p* = 0.318) or plasma (*p* = 0.192 and *p* = 0.466, respectively) was observed.

**Figure 3:**
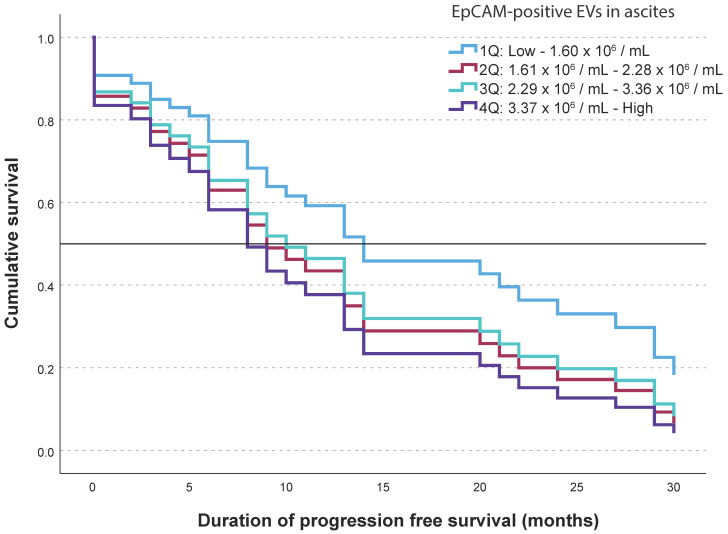
Kaplan–Meier survival curves show progression-free survival (PFS) according to EpCAM-positive EVs levels grouped in quartiles.

At the end of our study, 8 patients of 37 were alive and only 4 patients did not have detectable disease progression. Median follow-up time for survivors was 66 months (minimum 35 months and maximum 72 months). We looked at the relationship between total and EpCAM-positive EVs in ascites and plasma and PFS. Using proportional Cox survival regression, we found a significant association between EpCAM-positive EVs concentration in ascites and PFS (HR = 1.071, 95% CI: 1.007–1.138, *p* = 0.029). Higher EpCAM-positive EVs concentration in ascites was associated with shorter PFS (Figure 3).

No significant association between EpCAM-positive EVs in plasma and PFS was found, though. Also, there was no significant association between total EVs in ascites or plasma and PFS or total and EpCAM-positive EVs in ascites or plasma with OS.

Of the clinical characteristics that are presumably related with PFS and OS of patients with advanced HGSC, addition of bevacizumab (antivascular epithelial growth factor (anti-VEGF) antibody) in therapy and American Society of Anesthesiologists (ASA) score were evaluated. Also, we assessed CA125 value at the time of surgery, the time to CA125 normalization, and the CA125 elimination rate constant K (KELIM score, <1 or ≥1) in relation to PFS and OS. In our study group, PFS was strongly related with time to CA125 normalization, KELIM score and ASA score. Earlier CA125 normalization was strongly associated with longer PFS (HR = 10.683, 95% CI: 3.370–33.862, *p* < 0.001). Also, KELIM score ≥1 was associated with longer PFS (HR = 0.302, 95% CI: 0.105–0.874, *p* = 0.027). Higher ASA score on the other hand was connected to shorter PFS (HR = 3.004, 95% CI 1.294–6.974, *p* = 0.010). We found the same parameters associated with OS. Namely, shorter time to CA125 normalization (HR = 6.330, 95% CI: 2.310–17.342, *p* < 0.001) and KELIM score ≥1 (HR = 0.245, 95% CI: 0.067–0.893, *p* = 0.033) were strongly associated with longer OS and higher ASA score was associated with shorter OS (HR = 3.241, 95% CI: 1.331–7.891, *p* = 0.010). No significant relation between the level of CA125 at the time of surgery or addition of bevacizumab in therapy and PFS or OS was found. Neither total nor EpCAM-positive EVs in ascites and plasma were associated with time to CA125 normalization or KELIM score (<1 or ≥1). Summary of results is presented in Table 2.

## 3. Discussion

Platinum-resistant HGSC is still a fatal disease, so predictive biomarkers that could help with timely treatment adjustments and personalized approach are extensively being sought [3]. Tumor-derived EVs that contain tissue-specific signaling molecules (e.g., proteins, mRNA, and miRNA) and reflect the pathophysiological state of the cell of origin are such promising biomarkers [4,5]. In HGSC patients, EVs can be isolated from ascites and blood with minimally invasive liquid biopsy [4]. In vitro studies on ovarian cancer (OC) cell lines have shown that EVs play an important role in tumor progression and development of chemoresistance [4,7,8,9,10], but clinical studies of EVs’ potential to predict response to therapy and prognosis are still lacking. Interesting observations were described, though, in a preliminary study by Szajnik et al., who examined the association between exosomal protein content in plasma of 12 ovarian cancer patients and response to therapy. The exosomal protein levels variably changed during chemotherapy, suggesting that protein content of exosomes might be useful in predicting response to therapy of ovarian cancer patients. They also concluded that a selective molecular profile of tumor-derived EVs might be more informative [28].

Our study was conducted on pretreatment samples of ascites and plasma of 37 patients with advanced HGSC. All plasma samples were taken before primary surgery and ascites samples were taken at the beginning of primary cytoreductive or diagnostic operation. We used FT-FCM to determine total (calcein-labeled) and EpCAM-positive EVs concentration in paired ascites and plasma samples. First, we evaluated the correlation of total and EpCAM-positive EVs concentration between ascites and plasma. There was no correlation of total EVs concentration between ascites and plasma. The probable explanation is that total EVs concentration, especially in plasma, is influenced by various sources. On the other hand, we found a strong correlation of EpCAM-positive EVs between ascites and plasma. EpCAM-positive EVs are released from epithelial cells, but not from mesothelial cells that line the peritoneal cavity [14], so their presence in ascites is typical for epithelium-derived tumors. Our results also show a significantly higher concentration of EpCAM-positive EVs in ascites compared to plasma. Ascites is the pathological accumulation of fluid in the peritoneal cavity present in most (>90%) patients with advanced HGSC, though in variable quantity [29]. It contributes to disease spread and development of chemoresistance and is significantly associated with shorter progression-free survival (PFS) and overall survival (OS) [29]. It is an ideal medium for liquid biopsy; however, for clinical application of ascites-derived tumor biomarkers, standardization that would take into account the total volume of ascites or correlation with other molecular content is needed. So far, such protocols are lacking. In a study by Černe et al., where potential of soluble osteopontin (sOPN) in ascites as biomarker for epithelial ovarian cancer was studied, they found that concentration of sOPN in ascites was positively correlated with total volume of ascites and with total protein level [30]. In our study group, all patients (37, 100%) had ascites. Our protocol proposed the collection of 50 mL of ascites, which was possible in most (33/37, 89%) cases. In four cases, a smaller amount of ascites was collected due to procedural difficulties. Further research is needed to study the correlation of EpCAM-positive EVs levels with total ascites volume and set standards for clinical use, possibly by normalization of EVs levels to total ascites volume, total protein levels, or other important molecular content. However, in our study, a strong correlation of EpCAM-positive EVs levels between ascites and plasma was found.

Increased EpCAM-levels are typical of epithelium-derived tumors, and EpCAM overexpression in tumor tissue has been associated with poor prognosis in different carcinomas [14,15,16,17]. In ovarian cancer, studies on EpCAM overexpression in tumor tissue and survival gave contradictory results [18,19,20]—possibly because of the use of different anti-EpCAM antibodies, heterogeneous histological subtypes of epithelial ovarian tumors analyzed, and also different immunohistochemistry scoring methods [29]. Also, EpCAM is subjected to regulated proteolytic cleavage and the majority of antibodies that recognize EpCAM on cancer cells target the terminal (signal peptide) part of the extracellular domain that can be cleaved off [14]. Studies that used antibodies against EpCAM’s intracellular domain suggested that this cytoplasmic staining actually reflects tumor aggressiveness and is associated with prognosis [31,32]. In ascites and plasma, EpCAM is present in soluble form (sEpCAM) and on tumor-derived EVs [23,33]. EpCAM is also an attractive antigen for targeted therapies, and treatment of malignant ascites in cancer patients with EpCAM-positive tumors with the anti-EpCAM antibody catumaxomab has been approved already in 2009 [23]. However, most EpCAM-targeting therapies have not met expectations. Seeber et al. found that sEpCAM is able to neutralize the effect of catumaxomab in vitro [34] and later performed a study on ascites samples which found sEpCAM levels associated with poor survival of cancer patients treated with catumaxomab [23]. To our surprise, the relevance of EpCAM-positive EVs as predictive and prognostic biomarkers has been largely unexplored. Im et al. observed exosomal EpCAM expression in relation to response to standard chemotherapy [13]. They collected ascites samples of ovarian cancer patients before and after treatment and observed that increased levels of exosomal EpCAM and CD24 in samples after treatment were associated with nonresponding patients [13]. Their cohort only consisted of eight patients and was too small to obtain statistical significance. Also, the potential of exosomal EpCAM expression in pretreatment ascites samples to predict response to chemotherapy was not assessed.

In our study, 37 patients with advanced HGSC (FIGO stage III and IV) were included. Most, 22 (81%), were FIGO stage IIIC. Only 15 (41%) were eligible for treatment with primary cytoreductive surgery followed by adjuvant chemotherapy. Of the remaining 22 patients, 2 patients only received palliative chemotherapy, and one patient’s general condition was too poor for treatment with chemotherapy. A total of 19 patients were treated with neoadjuvant chemotherapy (NACT), followed by interval debulking surgery (IDS) in 16 cases (one patient declined IDS and two patients had disease progression during or soon after NACT and were not eligible for IDS). Bevacizumab was added to chemotherapy in 43% of all cases, although its addition did not affect PFS and OS. In our study group, median PFS was 10 months and median OS 29 months, which is inferior to data from the literature stating a median PFS from 16 to 21 months and a median OS from 32 to 57 months [35].

When patients with primary inoperable disease were compared with patients who had primary cytoreductive surgery regarding total and EpCAM-positive EVs levels in pretreatment ascites and plasma samples, no significant difference between the two groups was observed. Importantly, in our study population, there was also no difference in PFS or OS between the two groups.

On the other hand, we found a strong association between EpCAM-positive EVs concentration in ascites and PFS, regardless of treatment strategy. PFS was also related with time to CA125 normalization and KELIM score. The results of our study indicate that EpCAM-positive EVs levels in ascites of patients with advanced HGSC have potential as prognostic biomarkers. Since EpCAM is also present in a soluble form (sEpCAM) in body fluids, it is important that the chosen analytical method is able to discriminate between sEpCAM and EpCAM on EVs. sEpCAM monomers, per se, are too small (MW: 35 kDA) [14] to be detected by flow cytometry; however, if extracellular domains of EpCAM tended to aggregate and form multimers, they could affect the measurements. In our study, a detergent control was conducted. Detergent causes lysis of membrane-enclosed vesicles but does not affect sEpCAM. After treatment with detergent, EpCAM-positive EVs levels were drastically reduced, as shown in Figure S1 of the Supplementary Material.

Our research group recently studied characteristics of EVs from a HGSC cell line derived from a platinum-resistant patient in comparison to two cell lines from patients with platinum-sensitive disease. The chemoresistant cell line released more EpCAM-positive EVs. Also, a strong positive correlation between cellular EpCAM expression and the concentration of released EpCAM-positive EVs was observed [36]. On the other hand, the chemoresistant cell line did not release more total EVs. To further explore the potential of EpCAM-positive EVs to predict response to therapy and survival, we conducted this study on pretreatment samples of ascites and plasma of patients with advanced HGSC. The main limitation of our study was the small sample size. Patients also received different first-line therapies. Nonetheless, we found a strong correlation of EpCAM-positive EVs concentration between ascites and plasma and, most importantly, we found that EpCAM-positive EVs concentration in ascites, but not in plasma, is associated with shorter PFS of patients with advanced HGSC, regardless of treatment strategy. Total EVs concentration was not higher in patients with earlier recurrence, and no correlation in total EVs concentration was found between ascites and plasma. The findings of our study indicate that EpCAM-positive EVs levels in ascites of patients with advanced HGSC have potential as prognostic biomarkers for predicting early recurrence and thereby likelihood of more aggressive tumor biology and development of chemoresistance. Determination of EpCAM-positive EVs levels in ascites could also help select patients that would benefit from anti-EpCAM therapy and provide an improved personalized approach. For future research, especially on targeted therapy, evaluation of predictive value of EpCAM/epitope number per EV would also be interesting. More clinical studies on larger cohorts of patients with advanced HGSC are needed to test our results and further explore the potential of EVs as prognostic and predictive biomarkers.

## 4. Materials and Methods

### 4.1. Study Design

Patients with suspected or definite diagnosis of advanced HGSC were eligible for inclusion in this prospective cohort study. Primary surgery was performed at the Gynecological Department of University Medical Centre Ljubljana from October 2016 to August 2020. Patient data collected at baseline included age, menopausal status, body mass index (BMI), American Society of Anesthesiologists (ASA) score, and preoperative tumor marker CA125 value. On the day of primary surgery, blood samples were drawn and ascites samples were taken at the beginning of operation. When optimal cytoreductive surgery was expected, primary debulking surgery (PDS) was performed. Complete (R0) resection was considered where there was no macroscopic residual disease, optimal (R1) with less than 1 cm, and suboptimal (R2) with more than 1 cm of residual disease after cytoreductive surgery. When optimal cytoreduction was not expected, only diagnostic laparoscopy was performed. Pathology report included histologic type and residual disease status (R0, R1, or R2). In cases where the diagnosis of advanced stage HGSC was not confirmed with surgical assessment and/or histopathological examination, patients were excluded from the study.

Patients with PDS were treated with adjuvant chemotherapy (ACT), whereas patients with primary inoperable disease received primary neoadjuvant chemotherapy (NACT), followed by interval debulking surgery (IDS). Standard ACT/NACT regimen for HGSC is based on carboplatin in combination with paclitaxel. When a patient’s general condition did not allow this combination, carboplatin in monotherapy was applied. Bevacizumab (antivascular epithelial growth factor (anti-VEGF) antibody) was added to chemotherapy when the patient’s general condition allowed. For patients with IDS, secondary pathology report included chemotherapy response score (CRS). CRS is a histopathological scoring system that evaluates response to chemotherapy (CRS 1—no or minimal response, CRS 2—partial response, and CRS 3—complete or near-complete tumor response) (Santoro). For all patients, CA125 values at check-up during and after chemotherapy were collected. The modeled CA125 elimination rate constant K (KELIM score) was calculated with the CA125 longitudinal kinetics during the first 100 chemotherapy days.

Patients’ vital status was determined on 1 August 2023. Any missing data are clearly indicated.

Written informed consent was obtained from all patients eligible for study enrolment. The study was conducted in accordance with the Declaration of Helsinki and approved by the Republic of Slovenia National Medical Ethics Committee (KME 144/12/14).

### 4.2. Sample Collection

Samples were collected from fasting individuals (at least 12 h duration) to ensure that the blood was free of chylomicrons [37]. Blood was drawn before surgery using a 21-gauge needle. The first milliliters were used for routine clinical analyses. Blood samples (1.8 mL) for EV analyses were collected in BD vacutainer^®^ Citrate blood collection tubes with 3.2% buffered sodium citrate solution [38]. Ascites (50 mL) were aspirated into a sterile syringe at the beginning of operation and immediately transferred into a sterile conical tube. The maximum time interval between collection and preparation of samples was 30 min.

### 4.3. Sample Preparation and Storage

Preparation of blood and ascites samples for flow cytometric measurement was based on the “International Society on Thrombosis and Haemostasis Protocol” developed by Lacroix et al., which was adopted by the American Heart Association as methodological guidelines for EVs studies [39,40]. Later, this protocol was further validated, and all steps are described in detail in the publication “Protocol for measuring Concentration of extracellular vesicles in Human Blood Plasma with Flow Cytometry” [36]. This protocol or slightly different centrifugation protocols were used in several published EV biomarker studies [41,42,43,44]. There were two main reasons for choosing this protocol. Firstly, we had a very small volume of blood sample available. Secondly, we assumed that EpCAM-positive vesicles represent only a smaller part of total EVs, and so we did not want to reduce their number or completely remove them with a very rigorous isolation procedure.

Blood and ascites samples were processed by centrifugation with two consecutive steps of 2500× *g* for 15 min at room temperature to deplete cells and debris. After each centrifugation, a supernatant was collected at least 10 mm above the pellet. This procedure generates platelet-poor plasma samples (PPP; <10^4^ PLT/μL). Since ascites can also contain platelets, this also applies to ascites samples. Depletion of platelets and absence of hemolysis in samples were confirmed using routine clinical laboratory test [38,39,45].

Aliquots of plasma and ascites supernatants were frozen in nitrogen vapor in accordance with an adapted procedure for freezing sperm for artificial insemination [46] and stored at −80 °C until further analyses of EVs by flow cytometry. Only one freeze–thaw cycle was allowed for any sample.

Due to known problems with EVs analyses in complex clinical samples, especially in plasma, and because samples were analyzed without prior isolation of EVs, additional controls were conducted. We evaluated the influence of lipoproteins on our results by measuring the concentration of lipoproteins in our platelet-poor plasma (PPP) and ascites samples. Particle-enhanced immunonephelometry (Atellica^®^ NEPH 630 System, Siemens Healthineers, Erlangen, Germany) was used to determine concentration of Apolipoprotein B (chylomicrons, VLDL, IDL, and LDL) and Apolipoprotein A1 (HDL). Neither total EV (calcein-positive) nor EpCAM-positive EV concentration in plasma or ascites samples correlated with ApoA1 or ApoB concentrations (all *p* > 0.05). We also evaluated the influence of contaminating proteins in our samples on our results. Protein content was determined using the Bio-Rad Protein Assay (Hercules, CA, USA) according to the Bradford method. Total protein content did not correlate with total EV (calcein-positive) or EpCAM-positive EV concentration in plasma or ascites samples (all *p* > 0.05).

### 4.4. Fluorescence-Triggered Flow Cytometry (FT-FCM) of EVs

EVs were analyzed in accordance with our previously published protocol [34], with modifications that were necessary for clinical samples. The protocol was tested in our study on cell media from three different ovarian cancer cell lines, where FT-FCM results were validated using transmission electron microscopy, nanoparticle tracking analysis, and bead-based flow cytometry of EpCAM-positive EVs after anti-human EpCAM antibody-coated magnetic bead-based EV isolation.

In the present study, a flow cytometer (CytoFLEX, Beckman Coulter, Brea, CA, USA) was used to determine the concentration of calcein-positive and EpCAM-positive EVs in plasma and ascites samples of HGSC patients [36]. We performed assay control/proof of single EV detection in accordance with the recommendations included in a positional paper (MIFlowCytEV) developed and published by the EV Flow Cytometry Working Group [47], which is also referred to in the “MISEV 23 guidelines” prepared by the International Society for Extracellular Vesicles (ISEV) [48]. Fluorescence-based detection of EVs using FCM was employed to improve discernment of EVs from the background noise (Figure S1, Supplementary Materials). FT-FCM is also independent of particle size and refractive index [49]. Calcein-acetoxymethyl ester (AM) green (referred to as calcein) (Thermo Fisher Scientific, Waltham, MA, USA; #C3100MP) was used as a generic fluorescent marker for detection of EVs. Calcein AM is nonfluorescent until it passively enters EVs, where the lipophilic blocking groups are cleaved off by nonspecific esterase. As a result, calcein is trapped inside the EVs and emits a green fluorescent signal (emission maximum 516 nm) following excitation with a blue (488 nm) laser [50,51]. The advantage of calcein is differentiation of intact EVs from linearized membrane fragments of EVs and from other spherical particles that do not contain the required enzyme (e.g., apolipoproteins). The disadvantage of calcein is that the required enzyme may not always be present in sufficient quantity, even in intact EVs, leading to underestimation of total EVs concentration [52]. For EpCAM-positive EVs detection, phycoerythrin (PE)-conjugated anti-EpCAM (CD326) primary antibodies, clone 1B7 (Invitrogen/Thermo Fisher Scientific, Waltham, MA, USA, ZDA; #12-9326-42), or PE-conjugated isotype antibodies, mouse IgG1 kappa (Invitrogen/ Thermo Fisher Scientific, Waltham, MA, USA, ZDA; #12-1714-42), were used. Before staining, antibodies were centrifuged at 20,000× *g* for 30 min at room temperature to remove any fluorescent antibody aggregates, as described elsewhere [53]. Then, 20 µL of thawed PPP and ascites sample was incubated with 1.25 µL of PE-conjugated antibodies and kept in the dark for 2 h at room temperature. Due to some overlap between emission spectra of PE and calcein, which could lead to a false-positive signal, staining with calcein was performed on separate samples [54]. One hundred µL of calcein (10 µM) was added to 20 µL of sample and incubated in the dark for 30 min at room temperature. After incubation, 50 μL of paraformaldehyde (4%) was added to all samples, which were then diluted in DPBS to a count rate below 2000 events/s to prevent swarm detection [55,56].

Stained samples were measured within 1.5 h by flow cytometry using fluorescence triggering, timed at a slow flow rate for 120 s. Enumeration of EVs was performed using volumetric measurement (events/mL). Calibrating the sample flow rate was conducted following the CytoFLEX instructions by water weight difference during 18 min acquisition with a slow flow rate. To set up violet side scatter (VSSC) detection, we replaced the 450 nm filter with a 405 nm filter and appropriately labeled the detector as VSSC. For daily verification of the flow cytometer’s optical alignment and fluidics system, we used CytoFLEX Daily QC Fluorospheres (Beckman Coulter, #B53230) with settings optimized for EV detection.

Analysis of the acquired data was performed using CytExpert 2.3 software (Beckman Coulter, Brea, CA, USA). To calculate the number of events per mL for each sample, the dilution factor during sample preparation and staining was considered. Due to the detection limit of our flow cytometer, the EVs measured in this study predominantly correspond to ectosomes (microparticles), not to exosomes.

Additional experimental details are available in the supplementary section. We submitted all relevant data of our experiments to the EV-TRACK knowledgebase (EV-TRACK ID: EV240053). Additional information according to the MIFlow author checklist (Table S1) and MIFlowCyt-EV framework (Table S2) is provided in Supplementary Materials.

### 4.5. Statistical Analyses

All statistical analyses were performed using IBM SPSS Statistics, version 29 (IBM Corporation, Armonk, NY, USA). Continuous variables were described using the mean and the median with interquartile range (25–75%). Categorical variables were described using frequencies. Parametric ANOVA test and nonparametric Mann–Whitney test were used to compare the distribution of continuous variables among different patient groups. Pearson’s and Spearman’s rho correlation coefficient (ρ) were used to assess correlations between continuous variables. Proportional Cox survival regression was used to assess progression-free and overall survival. Progression-free survival was defined as the time from primary surgery to disease recurrence, and overall survival was defined as the time from primary surgery to death from any cause. All statistical tests were two-sided with the level of significance set to 0.05.

## Figures and Tables

**Table 1:** Patients’ characteristics.

Variables		Study Patients
Age	Years, median (25–75%)	66 (55–77)
ASA score	1, *n* (%)	0
	2, *n* (%)	19 (51%)
	3, *n* (%)	18 (49%)
FIGO stage	IIIA, *n* (%)	1 (2.7%)
	IIIB, *n* (%)	2 (5.4%)
	IIIC, *n* (%)	30 (81%)
	IVA, *n* (%)	1 (2.7%)
	IVB, *n* (%)	3 (8.1%)
Primary surgery	R0	5 (13.5%)
	R1	7 (18.9%)
	R2	3 (8.1%)
	inoperable	22 (59.4%)
Chemotherapy	Yes, ACT	15 (40.5%)
	Yes, NACT +/− ACT	19 (51.4%)
	Yes, palliative	2 (5.4%)
	No	1 (2.7%)
Bevacizumab	Yes, *n* (%)	16 (43.2%)
	No, *n* (%)	21 (56.8%)
CA125 preoperatively	kU/l, median (25–75%)	758 (460–1825)
CA125 normalization	Yes, *n* (%)	27 (73%)
	Days, median (25–75%)	71 (43–108)
	No, *n* (%)	10 (27%)
KELIM score	<1, *n* (%)	29 (80.6%)
	≥1, *n* (%)	7 (19.4%)
Progression/Relapse	Yes, *n* (%)	33 (89.2%)
	No, *n* (%)	4 (10.8%)
	Early < 12 months	20 (54.1%)
	Late ≥ 12 months or none	17 (45.9%)
PFS	Months, median (25–75%)	10 (5–22)
OS	Months, median (25–75%)	29 (19–56)

American Society of Anesthesiologists (ASA), International Federation of Gynecology and Obstetrics (FIGO), complete resection (R0), optimal resection (R1), suboptimal resection (R2), adjuvant chemotherapy (ACT), neoadjuvant chemotherapy (NACT), progression-free survival (PFS), overall survival (OS).

**Table 2:** Summary of statistics for EVs concentration in plasma and ascites, clinical characteristics, and survival of patients with advanced HGSC.

Observed Clinical Characteristics of Patients with Advanced HGSC	Total EVs Concentration in Plasma		Total EVs Concentration in Ascites	EpCAM-Positive EVs in Plasma	EpCAM-Positive EVs in Ascites		Test
Primary cytoreductive surgery vs. primary inoperable disease	0.805		0.318	0.098	0.262		Mann–Whitney
Overall survival	0.437		0.166	0.741	0.225		Cox regression
Progression-free survival	0.735		0.102	0.223	0.029 *		Cox regression
Time to CA125 normalization	0.102		0.113	0.125	0.066		Spearman correlation
		**Overall survival**		**Progression-free survival**			
Primary cytoreductive surgery vs. Primary inoperable disease		0.310		0.087		Cox regression	
Resection success at primary cytoreductive surgery (R0, R1, R2)		0.154		0.085		Cox regression	
Chemotherapy response score (CRS1, CRS2, CRS3)		0.948		0.690		Cox regression	
Addition of bevacizumab		0.934		0.919		Cox regression	
CA125 at the time of primary surgery		0.890		0.596		Cox regression	
Time to CA125 normalization		<0.001 *		<0.001 *		Cox regression	
KELIM score		0.033 *		0.027 *		Cox regression	
ASA score		0.010 *		0.010 *		Cox regression	

Results represent *p* values; * stands for *p* < 0.05, which was considered statistically significant. Complete resection (R0), optimal resection (R1), suboptimal resection (R2), no or minimal response to chemotherapy (CRS1), partial response (CRS2), compete or near complete tumor response (CRS3), the modeled CA125 elimination rate constant K (KELIM score), American Society of Anesthesiologists (ASA).

## Data Availability

The original contributions presented in the study are included in the article; further inquiries can be directed to the corresponding author.

## Supplementary Materials

The following supporting information can be downloaded at: https://www.mdpi.com/article/10.3390/ijms25126780/s1. Reference [57] is cited in the Supplementary Material.
