# Supplementary material for: Higher EpCAM-Positive Extracellular Vesicle Concentration in Ascites Is Associated with Shorter Progression-Free Survival of Patients with Advanced High-Grade Serous Carcinoma
Source: Int J Mol Sci. 2024 Jun 20;25(12):6780. doi: 10.3390/ijms25126780 (PMC11204144; doi:10.3390/ijms25126780)
Supplement: Supplementary file 1 [file ijms-25-06780-s001.zip › ijms-2984249-supplementary.pdf]

Supplementary Materials

Fluorescence-triggered flow cytometry (FT-FCM) of EVs

Fluorescence-triggered flow cytometry was performed using CytoFLEX, (Beckman Coulter, Brea, USA) to determine the concentration of calcein-positive and EpCAM-positive EVs in plasma and ascites samples of HGSC patients. Violet SSC (VSSC) is a third scatter-detection mode that is available to take the full advantage of the CytoFLEX sensitivity.

Supplementary Figure S1 legend

(A) Optimization of gains for VSSC, FSC and FITC with a mixture of polystyrene (PS) fluorescent beads of varied diameters to cover a major part of the theoretical EVs size range (100 nm, 300 nm, 500 nm, 900 nm). However, PS beads have a higher refractive index then EVs. Consequently, they underestimate EVs diameter. Thus, we used fluorescent silica beads (100 nm, 200 nm, 1000 nm), which have refractive index closer to EVs, to establish gate [1]. The size of 1000 nm silica beads roughly corresponds to 500 nm PS beads Using VSSC triggering, so the upper size limit for EV gate was set by detecting 1000 nm green-fluorescent silica beads (Kisker-biotech, #PSI-G1.0] and the lower size limit for EV gate was set by detecting 100 nm green fluorescent silica beads (Kisker-biotech, #PSI-G0.1]. (B) Dot plots showing fluorescence and size distribution of EVs from representative ascites and plasma samples. (C) Dot plots showing a fluorescence background of assay controls without EVs added: buffer only (DPBS) and buffer with reagents (calcein + DPBS, EpCAM antibodies + DPBS). (D) (E) Dot plots showing fluorescence of calcein-positive EVs from ascites (D) and plasma (E) sample. The threshold for fluorescence-triggering of calcein-positive events was set by unstained plasma or ascites EVs. Negative control was performed by staining samples with calcein at 4°C. Low temperature prevents the non-fluorescent calcein from being converted into the fluorescent form. The addition of 0.2% Triton X-100 (Sigma, #T8787) to calcein-stained EV samples for 20 minutes at room temperature resulted in near total disappearance of the EVs. (F) (G) The fluorescence signal from PE was used to trigger detection of EVs labelled with anti-PE-conjugated antibodies. The gate for PE was derived from measurements of corresponding isotype control antibodies. Negative control was performed with unstained EV samples. The addition of 0.2% Triton X- 100 to EpCAM stained EV samples for 20 minutes at room temperature resulted in near total disappearance of the EVs.

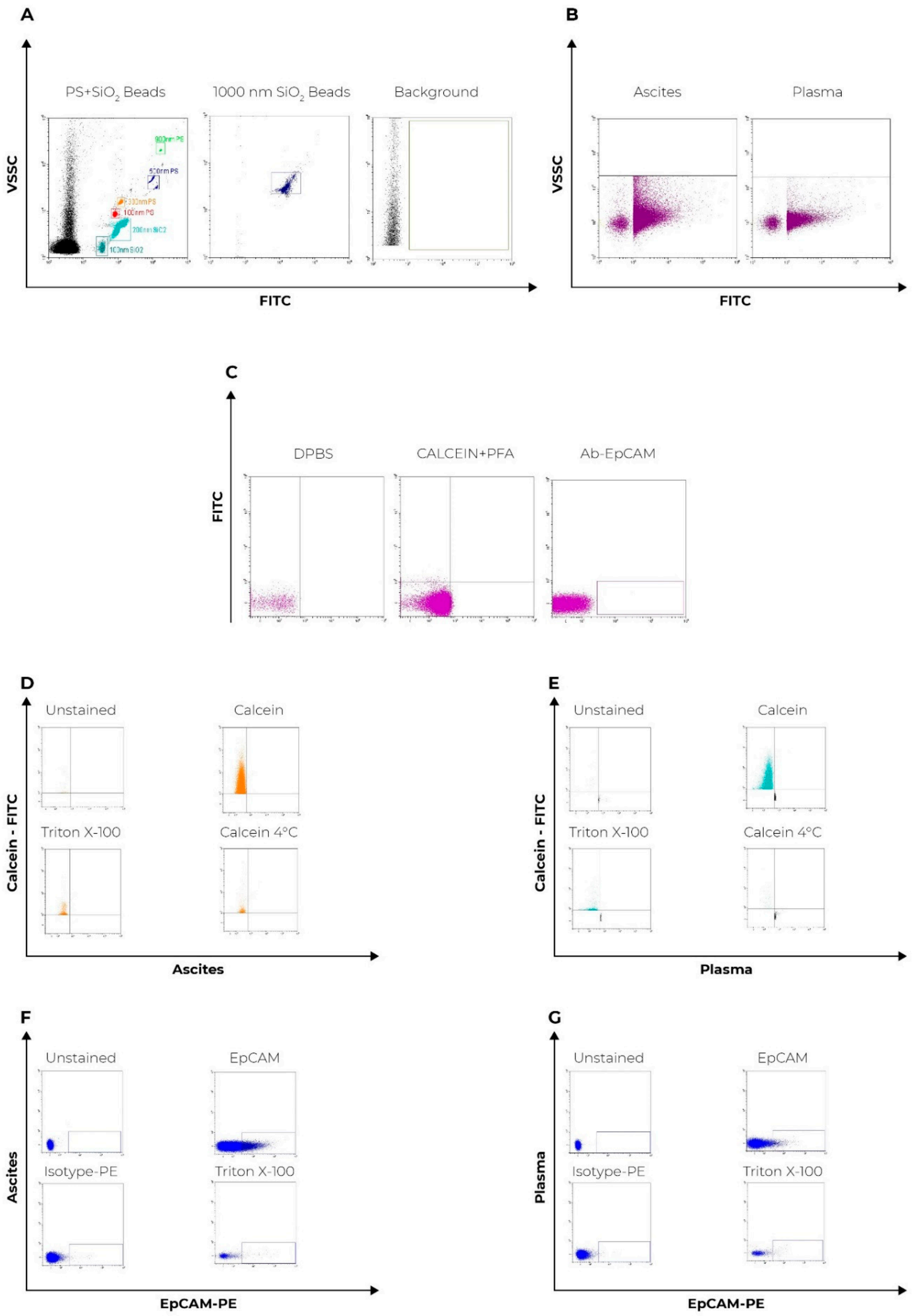

Figure S1. Analysis of total (calcein-positive) and EpCAM-positive EVs by fluorescence-triggered flow cytometry.

1. Parida BK, Garrastazu H, Aden JK, Cap AP, McFaul SJ. Silica microspheres are superior to polystyrene for microvesicle analysis by flow cytometry. Thromb Res. 2015 May;135(5):1000-6. doi: 10.1016/j.thromres.2015.02.011. Epub 2015 Feb 16. PMID: 25726425.

Supplementary Table S1. Author Checklist: MIFlowCyt-Compliant Items.

**Requirement**	**Please Include Requested Information**
1.1. Purpose	To evaluate the potential of total (calcein-positive) and EpCAM-positive EVs as prognostic biomarkers for advanced HGSC (ovarian cancer), that could help select patients who would benefit from anti-EpCAM targeted therapy and improve personalized approach.
1.2. Keywords	EpCAM, extracellular vesicles, HGSC (ovarian cancer), prognosis, biomarkers, personalized medicine
1.3. Experiment variables	-
1.4. Organization name and address	Institute of Pharmacology and Experimental Toxicology, Faculty of Medicine, University of Ljubljana, SI-1000 Ljubljana, Slovenia
1.5. Primary contact name and email address	Prof. dr. Katarina Černe, univ. dipl. biol. katarina.cerne@mf.uni-lj.si
1.6. Date or time period of experiment	June 2019 - October 2020.
1.7. Conclusions	The findings of our study indicate that EpCAM-positive EVs levels in ascites of patients with advanced HGSC have the potential as prognostic biomarkers for

	predicting early recurrence and thereby likelihood of more aggressive tumour biology and development of chemoresistance.
1.8. Quality control measures	Calibrating the sample flow rate was conducted following the CytoFLEX instructions by water weight difference during 18 minutes' acquisition with a slow flow rate.<br><br>For daily verification of the flow cytometer's optical alignment and fluidics system, we used CytoFLEX Daily QC Fluorospheres (Beckman Coulter, #B53230) with settings optimised for EV detection.<br><br>Assay controls.
2.1.1.1. (2.1.2.1., 2.1.3.1.) Sample description	On the day of primary surgery, blood samples were drawn and ascites samples were taken at the beginning of operation.
2.1.1.2. Biological sample source description	Patients with suspected or definite diagnosis of advanced HGSC were eligible for inclusion in this prospective cohort study.
2.1.1.3. Biological sample source organism description	Human.
2.1.2.2. Environmental sample location	

2.3. Sample treatment description	0.2% Triton X- 100 for 20 minutes at room temperature
2.4. Fluorescence reagent(s) description	Calcein-acetoxymethyl ester (AM) green (referred to as calcein) (Thermo Fisher Scientific; #C3100MP).<br>Phycoerythrin (PE)-conjugated anti-EpCAM (CD326) primary antibodies, clone 1B7 (Invitrogen/ Thermo Fisher Scientific, Waltham, ZDA; #12-9326-42). PE-conjugated isotype antibodies, mouse IgG1 kappa (Invitrogen/ Thermo Fisher Scientific, Waltham, ZDA; #12-1714-42)
3.1. Instrument manufacturer	Beckman Coulter.
3.2. Instrument model	CytoFLEX, B53010
3.3. Instrument configuration and settings	PE signals obtained upon excitation with the 488 nm laser were collected in the 585/42 band pas filter.<br>FITC signals obtained upon excitation with the 488 nm laser were collected in the 525/40 band pas filter.
4.1. List-mode data files	Are available upon request.
4.2. Compensation description	No compensation.
4.3. Data transformation details	No data transformation was applied.
4.4.1. Gate description	Isotype control. Unstained sample. 1000 nm green fluorescent silica beads (Kisker-biotech, #PSI-G1.0]. 100 nm

	green fluorescent silica beads (Kisker-biotech, #PSI-G0.1].
4.4.2. Gate statistics	The number of total events recorded in 120 seconds.
4.4.3. Gate boundaries	Images of gated plots can be found in supplementary materials.

Supplementary Table S2. MIFlowCyt-EV framework.

**Requirement**	**Please Include Requested Information**
1.1 Preanalytical variables conforming to MISEV guidelines	All relevant data of our experiments to the EV-TRACK knowledgebase (EV-TRACK ID: EV240053).
1.2 Experimental design according to MIFlowCyt guidelines	MIFlowCyt checklist can be found as part of the supporting information of this manuscript.
2.1 Sample staining details	Yes, described in Material and Methods.
2.2 Sample washing details	No washing.
2.3 Sample dilution details	Samples were diluted 25-fold – 100-fold.
3.1 Buffer-only controls	Yes, see Figure S1, B.
3.2 Buffer with reagent controls	Yes, see Figure S1, C.
3.3 Unstained controls	Yes, see Figure S1, D, E, F, G.
3.4 Isotype controls	Yes, see Figure S1, D, E, F, G.
3.5 Single-stained controls	N/A

3.6 Procedural controls	No.
3.7 Serial dilutions	Samples were diluted 25-fold – 100-fold.
3.8 Detergent-treated controls	Yes, sensitivity to Triton X-100; see Figure S1, D, E, F, G.
4.1 Trigger channel(s) and threshold(s)	Yes (VSSC, 525/40, 585/42). All relevant details can be found in Supplementary Materials.
4.2 Flow rate / volumetric quantification	Slow flow rate / yes.
4.3 Fluorescence calibration	No.
4.4 Scatter calibration	N/A
5.1 EV diameter/surface area/volume approximation	N/A
5.2 EV refractive index approximation	N/A
5.3 EV epitope number approximation	N/A
6.1 Completion of MIFlowCyt checklist	Yes, see table S1.
6.2 Calibrated channel detection range	Not provided.
6.3 EV number/concentration	Yes, see Figure 1, Table 2.
6.4 EV brightness	No.
7.1 Sharing of data to a public repository	Yes, all experimental details about the biological sample preparation can be found in EV-TRACK. All data fails are available upon reasonable request.
